# Treatment of the displaced femoral neck fracture, as reflected in *Acta Orthopaedica Scandinavica*: The rise and fall of internal fixation

**DOI:** 10.3109/17453671003635801

**Published:** 2010-03-31

**Authors:** Rolf Önnerfält

**Affiliations:** Department of Orthopedics, Lund University Hospital, LundSweden

## Introduction

The publication of Acta Orthopaedica Scandinavica started in1930, at the same time as “modern” treatment of the displaced femoral neck fracture was introduced by Sven Johansson (1932), who began his paper: “Scarcely any form of fracture has attracted so much discussion as fractures of the neck of femur, particularly those in the medial region”. Acta (with a change of name to Acta Orthopaedica in 2005) has devoted much space and interest to hip fracture, especially during the last 2 decades. Much has been published about the epidemiology in different geographical areas, for example, and rehabilitation. This report will focus on the treatment and outcome.

### The early years

Before nailing was introduced, the commonest treatment in the Scandinavian countries was according to Whitman or Whitman-LÖfberg, since Otto LÖfberg introduced and modified the method in Sweden. The fracture was reduced with extension, internal rotation, and abduction. Then the hip was immobilized in plaster for months. It is difficult to get a clear idea of the results. It seems that several fractures healed, but the mortality was high. LÖfberg practiced his method until 1946, when he retired from the Department of Surgery in MalmÖ (Lindqvist 1951). By then the orthopedists who practiced nailing had gradually taken over.

**Figure 1.**
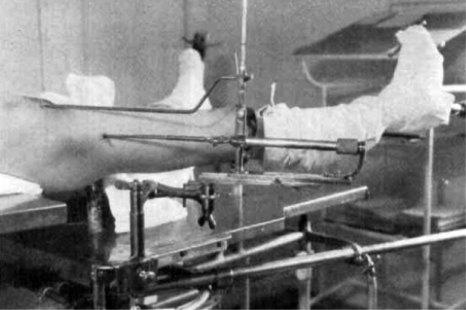
Patient on extension table with the targeting device assembled.

**Figure 2.**
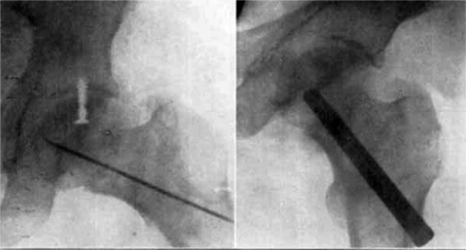
Guide pin in position and the final result.

**Figure 3.**
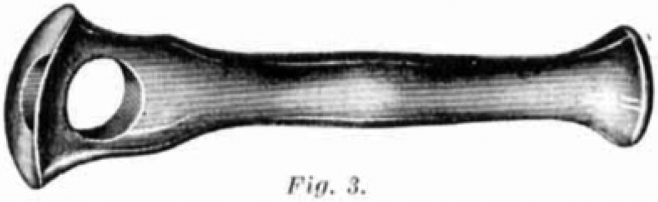
The compression hammer.

In 1931, Smith Petersen published in Archives of Surgery a new nail with 3 flanges, which was supposed to cause less pressure necrosis and to be rotationally stable. Smith Petersen believed that it was absolutely necessary to reduce the fracture openly. Sven Johansson (Gothenburg, Sweden), impressed by the design, aimed at a method using closed reduction: “Only by this means would the operation be relatively slight—so slight that, even having regard to the advanced age at which most of these fractures occur, it seemed to me to be justified” (Johansson 1932). He invented a technique resembling the one used today. He made a central canal in the Smith Petersen nail and invented a targeting device (Figure 1). After closed reduction and calculation with the help of external landmarks on the patient and a targeting device, a guide pin was introduced from the trochanter and into the femoral head. The position was checked with radiographs in two planes and if it was acceptable, the nail was “knocked in” (Figure 2). The position was again checked with radiographs and finally the fracture was compressed with a specially designed hammer (Figure 3). Dr Johansson presented his paper at the Nordic Orthopaedic Association, describing the first 9 cases: “…so encouraging to me, not least because of the advantage to the patients in the simplified aftertreatment, that I have not hesitated to already describe the method now for other surgeons and orthopaedists to try it”.

### More nails

**Figure 4.**
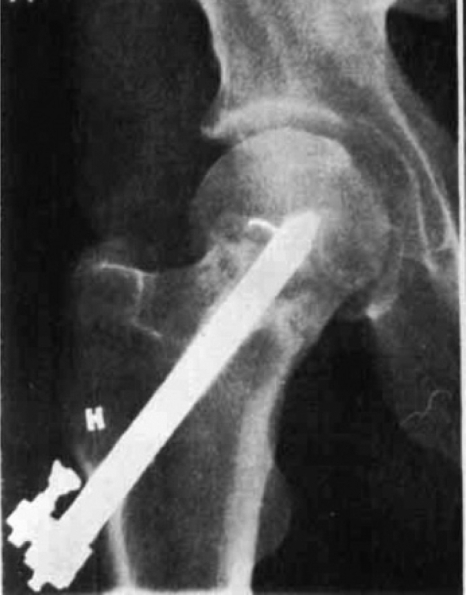
The spring-loaded nail. Mounted on the nail is a small compression plate, which was abandoned later.

**Figure 5.**
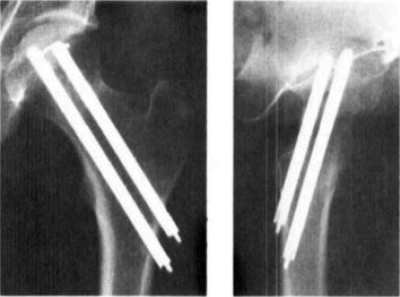
Hook pins.

**Figure 6.**
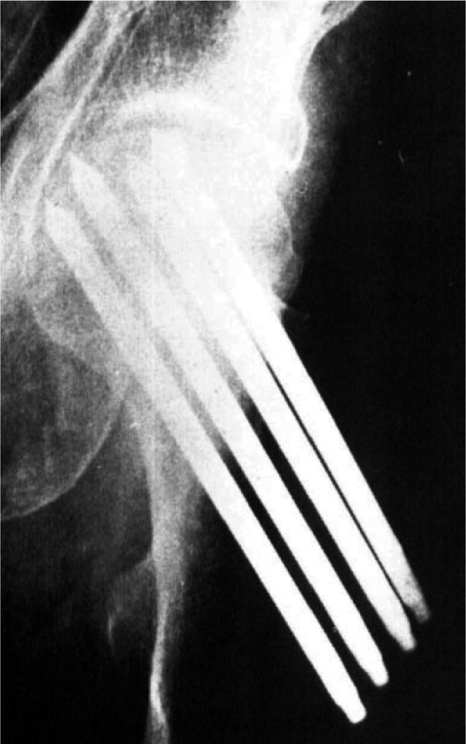
Multiple pinning.

Slipping of the nail was a common complication. The nail lost its hold and slipped out because of resorption of bone in the fracture area and around the nail. Rydell (1964) invented the “spring-loaded nail” (Figure 4). It had 4 flanges and was hammered in over a guide pin, which was removed and replaced by a spring pin. This pin had a curved end, which extruded through a hole in the nail in order to prevent slippage. The Rydell nail predominated until Hansson (1982) introduced the hook pin (Figure 5), simply a Rydell nail with flanges removed and with the same spring pin. It was primarily used for fixation of slipped capital femoral epiphysis. For neck fractures, 2 pins were used to prevent rotation of the head fragment. The pins were not hammered in but gently inserted through predrilled channels. A less gentle method was multiple pinning (Figure 6) (Kofoed and Alberts 1980). It was thought that the better stability obtained from multiple pins would facilitate revascularization of the femoral head.

### Sliding nail/screw-plate

**Figure 7.**
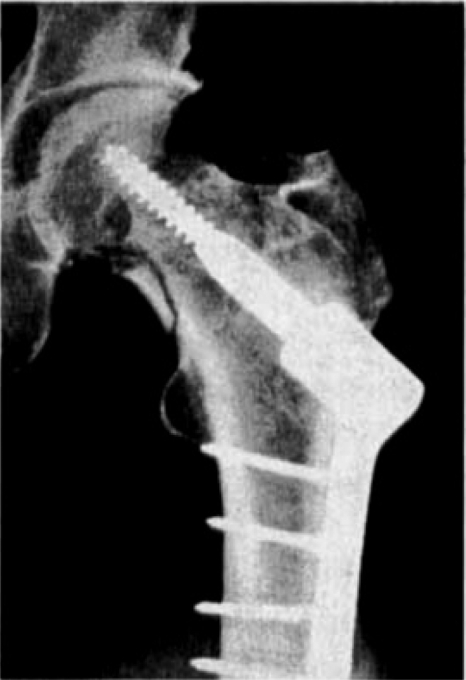
Sliding screw-plate.

**Figure 8.**
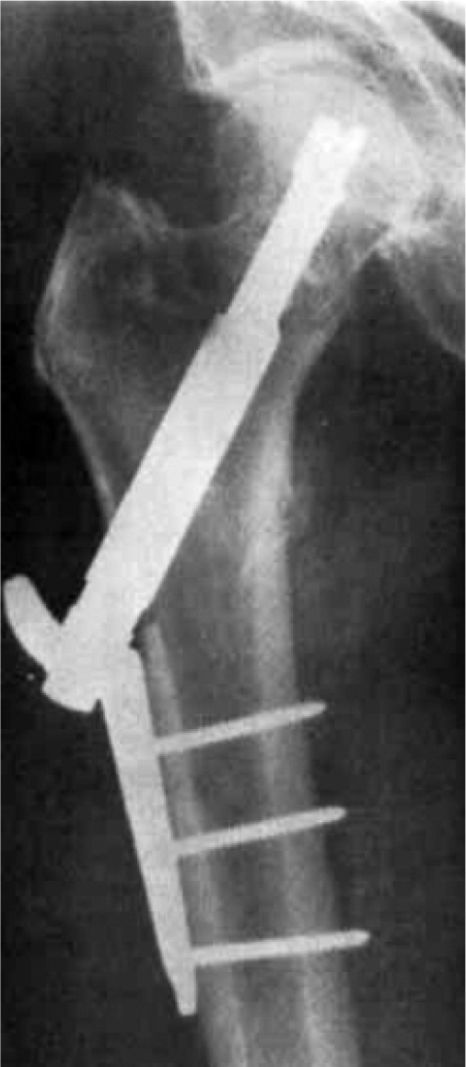
Sliding nail-plate.

Charnley was a forerunner also in the field of fracture treatment. He invented a sliding screw-plate for fixation of neck fractures (Figure 7). His paper from 1961 contains a detailed analysis of fixation and failure with his and contemporary methods of fixation. His device was a prototype of the sliding fixations used nowadays for trochanteric fractures. It appears that it was not widespread in the Scandinavian countries, but another sliding nail-plate (Figure 8) was used in Denmark (Frandsen and Jørgensen 1977).

### Screws

**Figure 9.**
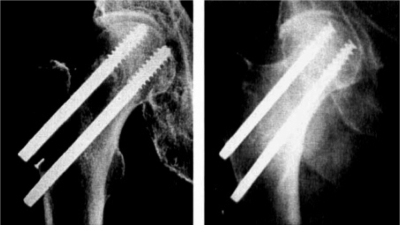
Uppsala (left) and von Bahr screws.

Several types of screws were introduced as an alternative to single-nail fixation. Some of them will appear later in this report, but the commonest were of Swedish design: the von Bahr screw and the Uppsala screw (Rehnberg and Olerud 1989). A guide pin was used for the Uppsala screw and the tip was self-tapping “to allow subchondral fixation” in the femoral head (Figure 9).

### Comparisons of outcome

With increasing options for internal fixation, it became necessary to compare the results of treatment. Was any one method superior?

#### Sliding nail/screw vs. pins/screws.

Frandsen and Andersen (1981) compared the Smith Petersen nail with sliding nail-plate. The patients were allocated to the 2 groups according to the date of admission. 83/131 fractures healed in the Smith Petersen group and 89/118 healed in the nail-plate group, a statistically significant difference. In a randomized trial, Madsen et al. (1987) compared sliding screw-plate with 4 ASIF cancellous screws. The rate of union (84%) was better in the screw group. Elmerson et al. (1995) compared hook pins with sliding screw-plate in a randomized study of 222 patients, and they found that the failure rate after 2 years was 38% with hook pins and 46% with screw-plate, which was a not statistically significant difference. Sørensen et al. (1992) randomized between sliding screw-plate and Gouffon screws. The study involved only 73 patients, but even so it clearly favored the use of a screw plate.

#### Rydell vs. Hansson.

The first comparison was conducted by Hansson (see StrÖmqvist et al. 1984). 5 selected surgeons operated on all patients with a neck fracture during one year, with 152 patients altogether. The patients were allocated according to even/uneven date of birth and were followed for 2 years. There were statistically significantly more complications in the Rydell group, and the difference was greatest in patients with a displaced fracture (23/32 as opposed to 12/36). Holmberg et al. (1990) conducted a similar but properly randomized study according to today’s standards. They reported early re-displacement or nonunion and did not find any statistically significant differences between the methods. This result was confirmed by Sernbo et al. (1990) in a larger randomized trial involving over 400 patients. They found similar rates of early displacement, nonunion, late segmental collapse, and salvage arthroplasty in the 2 groups.

### Uppsala screws vs. pins/screws.

Rhenberg and Olerud (1989) made a randomized comparison between Uppsala screws and von Bahr screws. There was a great difference between the screws regarding the common complications, and the Uppsala screw was better even after 1 month. Herngren et al. (1992) conducted a similar study comparing Uppsala screws and Hansson pins. In displaced fractures, re-displacement after 4 months was statistically significantly commoner with pins, and the overall result was slightly better with screws. Lagerby et al. (1998) randomized between 2 Uppsala screws and 3 Richards screws. They found no difference in complications and clinical results between the groups.

### Arthroplasty

As early as 1961, Charnley wrote: “It is probable that immediate prosthetic replacement of the femoral head after subcapital fractures of the neck of the femur, in senile patients, will be used more commonly in the future in preference to internal fixation. In senile patients there is much in favour of this policy, but we must not abandon attempts to improve internal fixation in the case of patients with an expectation of ten or more years of life. It is unfortunate that surgeons do not appear to have an agreed opinion on the frequency of failure of simple forms of internal fixation such as the Smith-Petersen nail.” By “senile”, he probably did not mean patients with cognitive impairment but elderly, fragile patients. His prediction proved to be right, but he could not have foreseen that it would take several decades to come true. During the period of efforts to improve internal fixation, primary arthroplasty almost disappeared. The philosophy was that since the fracture healed in most patients with a displaced neck fracture, the hip or the femoral head should be replaced only in patients who really needed it, i.e. after failure of fixation or because of femoral head necrosis.

#### Hemiarthroplasty.

**Figure 10.**
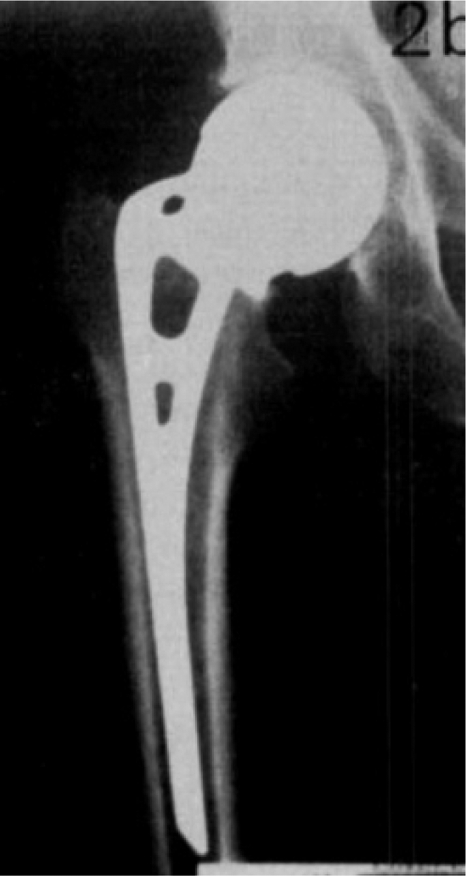
The Moore prosthesis.

Riska (1971) reported 107 primary replacements with the Moore prosthesis, most of them uncemented, because of subcapital fracture of the femoral neck (Figure 10). Riska argued that the patients were easier to mobilize, with less pain than after nailing. Steen Jensen and Holstein (1975) reported long-term results with the Moore prosthesis for 60 patients followed for 5(2.5–10) years. The prosthesis was used in elderly patients only (mean age 77 years). Cement was not used, which could explain why osteolysis around the stem and, above all, distal migration was common. Tillberg (1976) treated 163 patients with the same prosthesis and followed them for up to 9 years or until death (average 3 years). The mortality within 6 weeks was 9%. The functional result was good in 95% of surviving patients; 77% were free of pain and 93% managed ADL. Tillberg argued: “The usefulness of reposition and nailing of femoral-neck fractures in elderly patients is therefore open to question, and primary arthroplasty is recommended instead.”

Lindholm et al. (1976) reported similar results. Overgaard et al. (1991) used the Monk prosthesis, an uncemented bipolar hemiarthroplasty. They concluded that treatment with arthroplasty was advantageous because of the low complication rate.

It is strange indeed that the treatment with arthroplasty was almost completely abandoned, at least in Sweden, despite several reports of favorable results compared to internal fixation (Sernbo and Fredin 1993). Is there any explanation for why internal fixation became the only treatment for about 3 decades? Was it to save money, since internal fixation was cheaper (Søreide et al. 1980)? Was it to reduce the risks of treatment for old and fragile patients? Was it facilitate rehabilitation after a less invasive operation? In any case, after all efforts to improve internal fixation it became evident that the failure rate in displaced fractures was still not acceptable. Once more, arthroplasty became an interesting alternative to study.

### Internal fixation vs. arthroplasty.

In a small series comprising 47 patients, Jónsson et al. (1996) made a randomized comparison between hook-pin and a Charnley hip replacement. The patients were carefully selected: they came from their own home, were relatively healthy, and had normal walking ability before the fracture. After 1 and 2 years, fewer patients in the replacement group used outdoor walking aids and they were more likely to do their own shopping. Johansson et al. (2000) randomized 100 patients older than 75 years to fixation with 2 Olmed screws or total hip replacement with a Lubinus SP prosthesis. The mental state was classified and patients with cognitive dysfunction were included. Harris hip score was better in the arthroplasty group. Complications (mainly dislocation), mortality within 2 years, and reoperations in the screw group were different in patients with a normal mental state and those with a dysfunctional mental state. The authors concluded that hip replacement should be considered in mentally healthy, elderly patients with high functional demands. Parker and Pryor (2000) randomized 208 patients aged over 70 years between 3 cannulated screws and Austin Moore hemiarthroplasty. There were more re-admissions and reoperations after internal fixation, but the final functional outcome in the groups after 2–3 years was similar. RÖdén et al. (2003) randomized 100 patients aged over 70 years to treatment with either 2 von Bahr screws or Variokopf bipolar hemiprosthesis. Patients who did not remember their date of birth and home address were excluded. The Variokopf group had better walking capacity and less consumption of analgetics after 4 months. 34/53 patients had to be reoperated in the screw group and 3/47 in the prosthesis group. There was no difference in mortality between the groups after 2 or 5 years. A different approach was used by Bjørgul and Reikerås (2006). In a prospective study, they treated fractures with known bad prognosis after internal fixation (angulation of more than 30 degrees, comminution of the calcar, small proximal fragment (Alho et al. 1991)) with an Exeter hemiarthroplasty and fractures with a good potential for healing with Olmed screws. In spite of this selection of patients to internal fixation, the results were better in the Exeter group. Rogmark and Johnell (2006) performed a meta-analysis of 14 randomized studies comparing internal fixation and primary arthroplasty, and the conclusion was that arthroplasty should be used in most patients with a displaced femoral neck facture. Mortality was similar between the 2 groups at 30 days and 1 year.

### Register studies.

A report from the Norwegian Arthroplasty Register (Gjertsen et al. 2007) compared groups of elderly patients treated with total hip replacement: primary replacement in patients with an acute femoral neck fracture, secondary replacement because of sequelae after a neck fracture, and replacement because of osteoarthritis (OA). The 5-year survival of the hip replacement was 95% for primary replacements, 96% for secondary replacements, and 97% in patients with OA. There were increased risks of revision, especially during the first 6 postoperative months. Acute patients had an increased risk of revision compared to patients with OA. Hips with sequele had a higher risk of dislocation and periprosthetic fracture, but the risk of acetabular revision for loosening was lower. The report showed that hip replacement is a good treatment for acute fractures as well as late complications. The increased risks were small, and they would perhaps have been impossible to detect with fewer patients.

Most comparisons between internal fixation and arthroplasty have focused on complications and reoperations. Gjertsen et al. (2008) reported data from the Norwegian Hip Fracture Register about patient satisfaction, pain, and quality of life in patients operated on with internal fixation or a hemiarthroplasty. The results were better in the group treated with arthroplasty.

## Discussion

All arguments for internal fixation of displaced femoral neck fractures in elderly patients have proven to be wrong. Søreide et al. (1980) found that the total cost of prosthetic replacement was 1.6 times higher. The reason for this was that the cost of the initial hospital stay was 2.4 times higher after an arthroplasty. There was no classification of the fractures, and all neck fractures may have been included. According to the authors, the explanation for the difference in cost of hospital stay was greater postoperative morbidity in the arthroplasty group, but the mortality was the same. Rogmark et al. (2003) compared the costs of internal fixation with hook-pins and Charnley hip replacement or Charnley-Hastings hemiarthroplsty in a randomized study of 68 patients aged 70 years or more. Mentally deficient, bedridden, and institutionalized patients were excluded. The mean cost for a patient with internal fixation was 40% higher.

The opinion during the era of internal fixation monotherapy was that patients whose fracture healed without complications were winners; after healing, they could continue with a healthy hip. It was thought that the others did not lose very much; the complication could be easily treated with a hip or head replacement. This was a simplification of the problem and the clinical reality. At present, it is impossible to predict the healing for the individual patient. Also, fractures with seemingly good prognosis have a high failure rate (Bjørgul and Reikerås 2006). The losers had much to lose indeed. Usually it took several weeks and often many months of hip pain and severely reduced activity before the arthroplasty, and by then the general condition and the hip function had deteriorated. Blomfeldt et al. (2006) compared patients with primary hip replacement with patients who had had secondary replacement after failed fixation. The hip function was better after a primary replacement and the reduction of quality of life was more pronounced during the first year of treatment in the patients treated with fixation.

Should patients with cognitive deficiency, without any or with severely reduced walking capacity, or even bedridden patients be operated on with an arthroplasty, or is it “enough” with internal fixation? There is probably a widespread opinion that internal fixation is to be preferred. In the randomized comparisons, this category has usually been excluded. Johansson et al. (2000) reported more complications, mainly dislocations, after total hip replacements with a posterior approach. There is reason to believe that the risk of healing complications are similar and the complications as painful as in mentally healthy patients. It is difficult for patients with mental deficiency to complain. Perhaps they would also benefit from treatment with arthroplasty. Rogmark et al. (2002) studied primary arthroplasty in 103 patients aged 80 years or more. Included were patients with cognitive deficiency or prolonged confusion. Bedridden patients were excluded. The mean age was 87 (80–99) years. They were compared with a matched group treated with hook-pins. The cumulative failure rate after 1 year was 7% in the arthroplasty group and 36% after internal fixation. 2 hips became dislocated. With the best choice of implant and surgical approach, and skilled postoperative care adjusted to the special needs of this kind of patient, primary hemiarthroplasty could also be recommended for such patients with cognitive impairment.

Perhaps the subtitle—the rise and fall of internal fixation—is provocative. Internal fixation is still the first choice for non-displaced fractures. Also, young patients with a displaced fracture should be given the chance of healing. There is no clear upper age limit. The elderly should be treated with an arthroplasty—but with a hemiarthroplasty or a total hip replacement? Also, here the age limits are unclear and the routines appear to vary considerably. The more active the patient is, the more he/she will benefit from a total hip—even in the 70–80-year age group. There has been a rapid increase in the number of very old people, and there is a new population of survivors because of improved social and medical conditions creating new orthogeriatric problems. Hip fracture is one of the commonest and most costly diagnoses, and many new and old problems associated with it are still unsolved.

## References

[1] Alho A, Benterud JG, Ronningen H, Hoiseth A (1991). Radiographic prediction of early failure in femoral neck fracture. Acta Orthop Scand.

[2] Bjørgul K, Reikerås O (2006). Hemiarthroplasty in worst cases is better than internal fixation in best cases of displaced femoral neck fracture. Acta Orthop.

[3] Blomfeldt R, Törnqvist H, Ponzer S, Söderqvist A, Tidermark J (2006). Displaced femoral fracture: comparison of primary total hip replacement with secondary replacement after failed internal fixation. A 2-year follow-up of 84 patients. Acta Orthop.

[4] Charnley J (1961). The treatment of fractures of the neck of the femur by compression. Acta Orthop Scand.

[5] Elmerson S, Sjöstedt å, Zetterberg C (1995). Fixation or femoral neck fracture. A randomized 2-year follow-up study of hook pins and sliding screw plate in 222 patients. Acta Orthop Scand.

[6] Frandsen PA, Jørgensen F (1977). Osteosynthesis of medial fractures of the femoral neck by sliding nail-plate fixation. Acta Orthop Scand.

[7] Frandsen PA, Andersen PE (1981). Treatment of displaced fractures of the femoral neck. Smith-Petersen osteosynthesis versus sliding-nail-plate osteosynthesis. Acta Orthop Scand.

[8] Gjertsen J-E, Lie SA, Fevang JM, Havelin LI, Engesæter LB, Vinje T, Furnes O (2007). Total hip replacements after femoral neck fractures in elderly patients. Results of 8,677 fractures reported to the Norwegian Arthroplasty Register. Acta Orthop.

[9] Gjertsen J-E, Vinje T, Lie SA, Engesæter LB, Havelin LI, Furnes O, Fevang JM (2008). Patient satisfaction, pain and quality of life 4 months after displaced femoral neck fractures. A comparison of 663 fractures treated with internal fixation and 906 with bipolar hemirthroplasty reported to the Norwegian Hip Fracture Register. Acta Orthop.

[10] Hansson LI (1982). Osteosythesis with the hook-pin in slipped capital epiphysis. Acta Orthop Scand.

[11] Herngren B, Mörk-Petersen F, Bauer M (1992). Uppsala screws or Hansson pins for internal fixation o femoral neck fractures? A prospective study of 180 cases. Acta Orthop Scand.

[12] Holmberg S, Mattsson P, Dahlborn M, Ersmark H (1990). Fixation of 220 femoral neck fractures. A prospective comparison of the Rydell nail and the LIH hook pins. Acta Orthop Scand.

[13] Johansson S (1932). On the operative treatment of medial fractures of the neck of the femur. Acta Orthop Scand.

[14] Johansson T, Jacobsson S-A, Ivarsson I, Knutsson A, Wahlström O (2000). Internal fixation versus total hip arthroplasty in the treatment of displaced femoral neck fractures. Acta Orthop Scand.

[15] Jónsson BB, Sernbo I, Carlsson å, Fredin H, Johnell O (1996). Social function after cervical hip fracture. A comparison of hook-pins and total hip replacement in 47 patients. Acta Orthop Scand.

[16] Kofoed H, Alberts A (1980). Femoral neck fractures. 165 cases treated by multiple percutaneos pinning. Acta Orthop Scand.

[17] Lagerby M, Asplund S, Ringqvist I (1998). Cannulated screws for fixation of femoral neck fractures. No difference between Uppsala screws and Richards screws in an randomized prospective study of 268 cases. Acta Orthop Scand.

[18] Lindholm RV, Puranen J, Kinnunen P (1976). The Moore femoral-head prosthesis in fractures of the femoral neck. Acta Orthop Scand.

[19] Lindqvist I (1951). A survey of the late results in the treatment of fractura colli femoris medialis A. M. Whitman-Löfberg with particular reference to the clinical condition of the cases without union. Acta Orthop Scand.

[20] Madsen F, Linde F, Andersen E, Birke H, Hvass I, Poulsen TD (1987). Fixation of displaced femoral neck fractures. A comparison between sliding screw plate and four cancellous bone screws. Acta Orthop Scand.

[21] Parker MJ, Pryor GA (2000). Internal fixation or arthroplasty for displaced cervical hip fractures in the elderly. A randomised controlled trial of 208 patients. Acta Orthop Scand.

[22] Rehnberg L, Olerud C (1989). Fixation of femoral neck fractures. Comparison of the Uppsala and von Bahr screws. Acta Orthop Scand.

[23] Riska EB (1971). Prosthetic replacement in the treatment of subcapital fractures of the femur. Acta Orthop Scand.

[24] Rogmark C, Johnell O (2006). Primary arthroplasty is better than internal fixation of displaced femoral neck fractures. A meta-analysis of 14 randomized studies with 2,289 patients. Acta Orthop.

[25] Rogmark C, Carlsson å, Johnell O, Sernbo I (2002). Primry hemiarthroplasty in old patients with displaced femoral neck fracture. A 1-year follow-up of 103 patients aged 80 years or more. Aca Orthop Scand.

[26] Rogmark C, Carlsson å, Johnell O, Sernbo I (2003). Costs of internal fixation and arthroplasty for displaced femoral neck fractures. A randomized study of 68 patients. Acta Orthop Scand.

[27] Rydell N (1964). Osteosynthesis of medial collum fractures with the “spring loaded nail”. Acta Orthop Scand.

[28] Rödén M, Schön M, Fredin H (2003). Treatment of displaced femoral neck fractures. A randomized minimum 5-year follow-up study of screws and bipolar hemiposthesis in 100 patients. Acta Orthop Scand.

[29] Sernbo I, Fredin H (1993). Changing methods of hip fracture osteosynthesis in Sweden. An epidemiological enquiry covering 46 900 cases. Acta Orthop Scand.

[30] Sernbo I, Johnell O, Bååth L, Nilsson J-å (1990). Internal fixation of 410 cervical hip fractures. A randomized comparison of a single nail versus two hook-pins. Acta Orthop Scand.

[31] Steen Jensen J, Holstein P (1975). A long term follow-up of Moore arthroplasty in femoral neck factures. Acta Orthop Scand.

[32] Strömqvist B, Hansson LI, Nilsson LT, Thorngren K-G (1984). Two-year follow-up of femoral neck fractures. Comparison of osteosynthesis methods. Acta Orthop Scand.

[33] Søreide O, Alho A, Reitti D (1980). Internal fixation versus endoposthesis in the treatment of femoral neck fractures in the elderly. A prospective analysis of the comparative costs and consumption of hospital resources. Acta Orthop Scand.

[34] Sørensen JL, Varmarken J-E, Bømler J (1992). Internal fixation of femoral neck fractures. Dynamic Hip and Gouffon screws compared in 73 patients. Acta Orthop Scand.

[35] Tillberg B (1976). Treatment of fractures of the femoral neck by primary arthroplasty. Acta Orthop Scand.

[36] Overgaard S, Toftgaard Jensen T, Bonde G, Mossing NB (1991). The uncemented bipolar hemiarthroplsty for displaced femoral neck fractures. Acta Orthop Scand.

